# The role of NLRP3 inflammasome in age-related macular degeneration: mechanisms and therapeutic prospects

**DOI:** 10.3389/fnagi.2026.1817987

**Published:** 2026-07-16

**Authors:** Meijiao Zhu, Weihong Yu

**Affiliations:** 1 Department of Ophthalmology, Peking Union Medical College Hospital, Chinese Academy of Medical Sciences and Peking Union Medical College, Beijing, China; 2 Eight-Year MD Program Peking Union Medical College (PUMC) Hospital, PUMC & Chinese Academy of Medical Sciences, Beijing, China

**Keywords:** age-related macular degeneration, inflammation, NLRP3 inflammasome, pyroptosis, retinal pigment epithelial cells, targeted therapy

## Abstract

Age-related macular degeneration (AMD) is a fundus oculi disease that progressively impairs the central vision of patients. To date, its pathogenesis has not been fully elucidated, and therapeutic options for dry AMD remain limited. Recently, chronic low-grade inflammation has been recognized as an important pathogenic factor in various neurodegenerative diseases, including AMD. The NLRP3 inflammasome, a key component of the innate immune system, has emerged as a critical integrator of retinal stress signals. This review first delineates the molecular architecture and activation modalities of the NLRP3 inflammasome, encompassing canonical, noncanonical, and alternative pathways, as well as its downstream cell death programs, with a particular focus on pyroptosis and PANoptosis. We describe how AMD-associated danger signals converge on NLRP3 inflammasome activation within distinct retinal cell populations and discuss how cell-type-specific NLRP3 responses differently shape retinal homeostasis, degeneration, and neovascularization. We further summarize current evidence indicating that the pathological consequences of NLRP3 activation vary across AMD progression, from amplification of chronic inflammation in early and intermediate AMD to promotion of retinal atrophy in geographic atrophy and angiogenic signaling in neovascular AMD. Finally, we evaluate emerging therapeutic strategies targeting the NLRP3 pathway and discuss the major translational challenges related to cell-type and disease-stage specificity, retinal delivery, and long-term safety. By integrating retinal triggers, cellular responses, senescence-associated inflammation, inflammatory cell death, disease phenotypes, and therapeutic opportunities into a unified framework, this review provides a comprehensive perspective on the role of NLRP3 inflammasome signaling in AMD pathogenesis and treatment.

## 1 Introduction

Age-related macular degeneration (AMD) is an eye disease that progressively impairs central vision and is the leading cause of blindness in individuals over 50 in developed countries. It is projected that the number of global AMD patients will increase to 288 million by 2040 (Wong et al., 2014). In China, the number of AMD patients has been continuously growing due to population aging, and the prevalence in people over 70 years old has reached 20.2% (Ye et al., 2014). Yet, the exact etiology and mechanism of AMD remain unclear, likely involving genetic factors, environmental influences, chronic photo damage, trophic disorders, metabolic disturbance, and so on (Heesterbeek et al., 2020).

Early and intermediate-stage AMD is characterized by deposits accumulated between the retinal pigment epithelium (RPE) layer and Bruch’s membrane called drusen, or a more diffuse form of deposits present in the subretinal space called subretinal drusenoid deposits (SDDs) (Zweifel et al., 2010). Drusen are mainly composed of lipids, proteins, and minerals, among which lipids account for at least 40%, mostly cholesterol and phosphatidylcholine (Wang et al., 2010). Proteins such as vitronectin, serum albumin, apolipoproteins E and B, complement proteins like C3, C5, and C5b-9, factor X, and Aβ peptides, as well as carbohydrates and minerals like zinc accumulation, are also important components (Crabb et al., 2002; Malek et al., 2003; Mullins et al., 2000; Anderson et al., 2004; Mullins and Hageman, 1999; Lengyel et al., 2007). As the disease progresses, drusen grow in size and number, accompanied by the pathological changes of the RPE layer, including the detachment and migration of RPE cells into the neuroretina. Late-stage AMD has two distinct forms: geographic atrophy (GA), the late-stage atrophic manifestation of AMD, and neovascular AMD (nAMD, also known as wet AMD). GA is defined by the atrophy of photoreceptors, RPE, and choriocapillaris due to cell death. The areas of GA can enlarge over time, with the loss of central vision. Neovascular AMD involves the abnormal growth of new vessels stimulated by vascular endothelial growth factor (VEGF), which invade the subretinal space and sub-RPE space, resulting in macular neovascularization (MNV) (Fleckenstein et al., 2021). In the exudative stage, the vessels leak or rupture, leading to subretinal fluid accumulation, accompanied by the destruction of photoreceptors, RPE, and choriocapillaris, and finally the formation of fibrosis scars, which also damage the central vision (Liu D. et al., 2023). To date, injection of anti-VEGF agents such as Ranibizumab, Bevacizumab, and Aflibercept into the vitreous cavity has been regarded as the first-line therapy of wet AMD, and it can effectively reduce the blindness rate (Rosenfeld et al., 2005, 2006; Heier et al., 2012). However, approximately half of the treated individuals still lose vision after 5–7 years (Daniel et al., 2018), and treatment options for dry AMD, particularly its advanced stage – GA, still remain limited. Thus, new therapies for AMD need to be explored. Since chronic low-level inflammation is causative in various neurodegenerative diseases, including AMD (Nita et al., 2014), treatments targeting the innate immune system come into sight. Among the innate immune pathways implicated in AMD, the NLRP3 inflammasome has emerged as a pivotal inflammatory platform that can be activated by multiple pathogenic stimuli in the retina and subsequently exacerbates ocular pathologies by amplifying retinal inflammation (Brandli et al., 2024). Increasing experimental and clinical evidence has demonstrated that aberrant NLRP3 inflammasome activation contributes to both dry and wet AMD progression (Tarallo et al., 2012; Marneros, 2023; Li et al., 2023; Ortega et al., 2025), highlighting its potential as a promising therapeutic target. Therefore, this review delineates the molecular mechanisms underlying NLRP3 inflammasome activation, elucidates its pathogenic roles in AMD, and evaluates candidate therapeutic strategies targeting this pathway.

## 2 NLRP3 inflammasome

To fully grasp how NLRP3 drives the multifaceted pathologies of AMD, it is first essential to dissect the precise molecular architecture and activation modalities of the NLRP3 inflammasome. Although NLRP3 is historically characterized in standard immune cells like macrophages, evidence has indicated that the retinal microenvironment possesses unique endogenous stressors that exploit these diverse pathways in a cell-specific manner (Tong and Wang, 2020; Brandli et al., 2024). Therefore, this section provides a comprehensive structural and mechanistic framework of NLRP3 activation, serving as a prerequisite to understanding the diverse pathological roles in AMD.

### 2.1 Pattern recognition receptors (PRRs) and the structure of NLRP3

Inflammasomes, a group of intracellular multimeric protein complexes, function as molecular platforms for the activation of the caspase family in the innate immune system, particularly caspase-1. Canonical inflammasomes typically comprise three core components: a sensor, an adaptor, and an effector. Within this framework, the NLRP3 protein, a member of pattern recognition receptors (PRRs), acts as a sensor of harmful stimuli and the starting point of NLRP3 inflammasome assembly.

Pattern recognition receptors (PRRs) are germline-encoded innate immune sensors that recognize pathogen-associated molecular patterns (PAMPs) derived from invasive foreign pathogens and damage-associated molecular patterns (DAMPs) released from stressed, damaged, or senescent cells (Takeuchi and Akira, 2010). Once stimulated, PRRs initiate the downstream intracellular signal transduction, which subsequently induces the following inflammatory responses to coordinate host defense and tissue homeostasis. Among the major PRR families, NOD-like receptors (NLRs) are particularly important in sterile inflammatory diseases because they primarily serve as cytosolic sensors of endogenous danger signals (Velloso et al., 2019). This feature is especially relevant in AMD, where chronic retinal degeneration is largely driven by sterile inflammation rather than exogenous infection.

NOD-, LRR-, and pyrin domain-containing protein 3, namely NLRP3, belongs to the NLR protein family. Structurally, the NLRP3 protein consists mainly of three domains: an N-terminal pyrin domain (PYD) that recruits the adaptor protein apoptosis-associated speck-like protein (ASC), a central nucleotide-binding oligomerization (NOD or NACHT) domain mediating ATP hydrolysis, and a C-terminal leucine-rich repeat domain (LRR) that is involved in self-inhibition (Xu and Núñez, 2023). Recent structural studies have proposed that murine NLRP3 monomers can spontaneously form a cage-like oligomer through LRR interactions, which shields the PYD and NACHT domains from downstream signaling partners (Schroder and Coll, 2021). The fish-specific NACHT-associated domain (FISNA) that appears to act as the conformational switch can sense ionic flux and release the oligomer into an active single-ring structure (Tapia-Abellán et al., 2021). This conformational plasticity partly underlies the tightly regulated nature of NLRP3 activation, ensuring that inflammasome assembly occurs only upon appropriate stimulation. Dysregulation of this activation switch may lead to the persistent inflammasome activity observed in chronic retinal inflammatory conditions. More importantly, the dynamic transition between inactive and active states may offer a structural rationale for designing small-molecule inhibitors that stabilize the cage conformation.

### 2.2 The activation of the NLRP3 inflammasome

**FIGURE 1:**
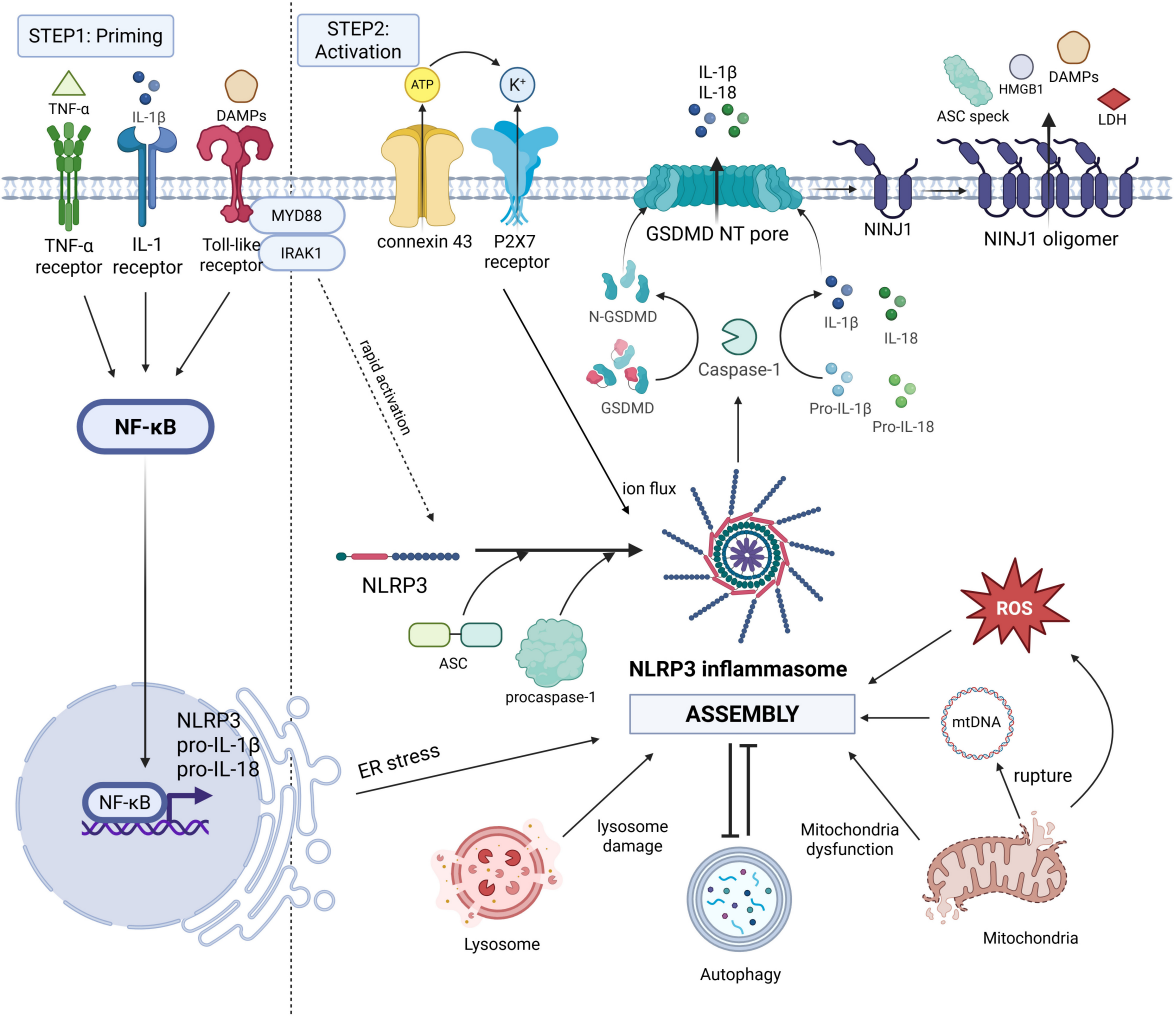
Mechanism of canonical NLRP3 inflammasome activation. In general, the canonical pathway requires two steps: priming and activation. The priming step is relatively complicated. Ligands (such as LPS, HMGB1, TNF-α, and IL-1) can trigger TLRs and cytokine receptors. Then, the expression of NLRP3, pro-IL-18, and pro-IL-1β is promoted via the activation and translocation of the transcription factor NF-κB. Additionally, post-translational modification can regulate the NLRP3 inflammasome. Following the priming step, the activation step is initiated by various PAMPs and DAMPs, such as ionic flux, mitochondrial dysfunction, ROS, lysosomal damage, and ER stress. The rupture of mitochondria not only releases mtDNA and mtROS, but also exposes cardiolipin that is originally located on the mitochondrial inner membranes, which all contribute to activation. In addition, autophagy and the NLRP3 inflammasome mutually suppress each other. ASC binds NLRP3 and recruits procaspase-1 via PYD and CARD domains respectively, thus accomplishing NLRP3 inflammasome assembly. Caspase-1 is formed by self-cleavage and subsequently processes pro-IL-18 and pro-IL-1β into their mature forms, IL-18 and IL-1β. GSDMD-NTs, also cleaved by caspase-1, oligomerize and form pores on the cell membrane, through which small molecules such as cytokines and ions can be released. As the membrane integrity declines, membrane protein NINJ1 also oligomerizes and promotes the plasma membrane rupture, causing the release of large molecules, including DAMPs, HMGB1, and LDH. Both pro-inflammatory cytokines and DAMPs can further propagate and aggravate inflammation. This figure was created in https://BioRender.com .

NLRP3 inflammasome activation can proceed through canonical, noncanonical, and alternative pathways. In general, the canonical pathway requires a two-step cascade comprising priming and activation. The priming step is relatively complicated. Various ligands can trigger Toll-like receptors (particularly TLR2 and TLR4), cytokine receptors (such as IL-1 receptor, TNF-α receptor), and NLRs (such as NLR1 and NLR2) (Franchi et al., 2009; Huang et al., 2021). In AMD, this priming process may be chronically sustained by persistent low-grade inflammatory stimuli such as oxidative stress, drusen components, and senescence-associated secretory factors within the aging retina (Kauppinen et al., 2012; Doyle et al., 2012; Brandstetter et al., 2015a,Liu et al., 2013; Wang et al., 2024). These local stimuli promote the expression of NLRP3, pro-IL-18, and pro-IL-1β via the activation and translocation of the transcription factor NF-κB. Additionally, the TLR downstream adaptor proteins MyD88 and IL-1R-associated kinase 1 (IRAK-1) can directly regulate the activation of NLRP3 (Lin et al., 2014) (Figure 1).

Post-translational modifications such as ubiquitination, deubiquitination, phosphorylation, dephosphorylation, and SUMOylation can also regulate the priming step of the NLRP3 inflammasome (O’Keefe et al., 2024). Although NLRP3 ubiquitination has been explored in RPE cells (Huang et al., 2020), whether these PTM patterns undergo pathological shifts during retinal aging and contribute to disease progression remains an important unresolved question in AMD research.

Following the priming step, a wide variety of PAMPs and DAMPs can trigger the activation step. These stimuli induce multiple molecular and cellular events, such as ionic flux (K^+^/Cl^–^ efflux, Na^+^ influx, and Ca^2+^ mobilization), mitochondrial dysfunction, the release of reactive oxygen species (ROS) and mitochondrial DNA (mtDNA), lysosomal damage, and ER stress (Muñoz-Planillo et al., 2013; Tang et al., 2017; Shimada et al., 2012; Murakami et al., 2012; Abais et al., 2015; Zhou et al., 2011; Hornung et al., 2008; Menu et al., 2012). Notably, many of these activating signals are markedly enriched in the aging retina and degenerating RPE cells. In addition, autophagy that removes DAMPs, damaged proteins, and organelles restricts NLRP3 activation. Reciprocally, NLRP3 inflammasome negatively controls the autophagic process through a caspase-1-dependent pathway (Jabir et al., 2014; Biasizzo and Kopitar-Jerala, 2020). Given that impaired autophagy and lysosomal dysfunction are well-documented features of AMD pathology (Kaarniranta et al., 2023), defective intracellular clearance represents a plausible mechanistic link between RPE aging and sustained NLRP3 inflammasome activation.

Recent studies further suggest that NLRP3 inflammasome activation is not merely a biochemical signaling cascade, but also a spatially organized biophysical process involving biomolecular condensates and liquid–liquid phase separation (LLPS)-like events. Recent work by Zou et al. has proposed LLPS as a convergent initiating mechanism that may integrate diverse activating stimuli into a unified inflammasome response (Zou et al., 2025). This LLPS model may provide a conceptual framework for understanding how structurally diverse AMD-associated stressors, including oxidative stress, mitochondrial dysfunction, lysosomal damage, and extracellular danger signals, culminate in a common NLRP3 activation pathway. However, whether LLPS-like dynamics occur in retinal cells during AMD progression remains to be determined.

In a recent structural model, the dispersed trans-Golgi network (dTGN) recruits the NLRP3 via phosphatidylinositol-4-phosphate (PtdIns4P). Then, dTGN serves as a scaffold, where the NACHT domain binds NIMA-related kinase 7 (NEK7), thereby stabilizing the complex and promoting the subsequent recruitment of ASC (Chen and Chen, 2018). ASC bridges NLRP3 and procaspase-1 through homotypic PYD–PYD and CARD–CARD interactions, leading to the formation of the NLRP3 inflammasome complex. Subsequently, procaspase-1 undergoes self-cleavage to form active caspase-1. Caspase-1 then proteolytically processes pro-IL-18 and pro-IL-1β into their mature forms, IL-18 and IL-1β (Yu and Finlay, 2008). Caspase-1 can also specifically cleave gasdermin D (GSDMD) and release its N-terminal fragment (GSDMD-NT), which functions as a pore-forming protein that executes pyroptosis (Ding et al., 2016).

While the canonical pathway is caspase-1-dependent, the noncanonical pathway depends on caspase-11 in mice and its human orthologs caspase-4 and caspase-5. Similar to caspase-1, caspase-4/5/11 can also cleave GSDMD to induce small molecule leakage and pyroptosis (Casson et al., 2015). This GSDMD-mediated membrane permeabilization, in turn, triggers K^+^ efflux, which acts as a secondary signal to promote the assembly and activation of the canonical NLRP3 inflammasome (Huang et al., 2021). Furthermore, recent work has demonstrated that the orphan receptor Nur77, which binds mitochondrial DNA and LPS, is required for activating the noncanonical NLRP3 inflammasome (Zhu et al., 2023). The contribution of the noncanonical inflammasome pathway to AMD has also been demonstrated. Kerur et al. showed that RPE degeneration in AMD is driven by a cGAS-dependent noncanonical inflammasome pathway, with elevated caspase-4 and cGAS levels confirmed in human GA eyes (Kerur et al., 2018).

The alternative pathway is devoid of any typical feature of classical inflammasomes. The alternative inflammasome is activated via the TLR4-TRIF-RIPK1-FADD-CASP8 signaling cascade upstream of NLRP3 (Gaidt et al., 2016). Moreover, apolipoprotein C3 has also been shown to induce alternative inflammasome activation through facilitating the heterodimerization of TLR2 and 4 and activating caspase-8 in human monocytes (Zewinger et al., 2020). Given that apolipoproteins, including Apo E and Apo C, are major structural constituents of sub-RPE drusen deposits, these findings raise the intriguing possibility that lipid accumulation may engage alternative inflammasome signaling within RPE cells or the sub-retinal space. This pathway therefore provides a potential mechanistic link between dysregulated lipid metabolism and chronic NLRP3-mediated inflammation in AMD, although direct evidence in retinal cells remains limited.

### 2.3 The NLRP3 inflammasome in pyroptosis and beyond

Beyond cytokine maturation and inflammatory signaling, NLRP3 inflammasome activation can also determine cellular fate through several forms of regulated cell death. This aspect is particularly relevant to AMD, where progressive RPE degeneration and photoreceptor loss constitute the central pathological features of late-stage atrophic AMD. Therefore, understanding how NLRP3 activation links inflammation to cell death provides an important mechanistic basis for interpreting retinal degeneration in AMD.

#### 2.3.1 Classical pyroptosis: the caspase-1/GSDMD axis

Classical pyroptosis is typically mediated by canonical inflammasomes (including NLRP3, NLRC4, AIM2, and others) (Pandey et al., 2025), with NLRP3 being the best-characterized in the context of AMD (Celkova et al., 2015). Pyroptosis is a form of regulated cell death characterized by cell swelling, pore formation, membrane rupture, and release of cell contents, which is important for the clearance of pathogens and the propagation of inflammation (Rao et al., 2022; Hur and Steinberg, 2025). The pore-forming protein GSDMD-NT, formed in the NLRP3 inflammasome pathway, acts as a key executor protein of pyroptosis. Following cleavage, GSDMD-NT oligomerizes and inserts into the plasma membrane, forming a pore with an inner diameter of 10–14 nm (Ding et al., 2016). Small molecules such as pro-inflammatory cytokines (prominently IL-18 and IL-1β), ions (prominently K^+^ efflux), and small cytoplasmic proteins can be released through the pores (Evavold et al., 2018). As GSDMD-NT pores disrupt the plasma membrane integrity and increase its permeability, recent studies have demonstrated that ninjurin-1 (NINJ1) actively mediates the following plasma membrane rupture, causing the release of large molecules, including DAMPs, high-mobility group box 1 (HMGB1), and lactate dehydrogenase (LDH) (Kayagaki et al., 2021). The resulting secretion of pro-inflammatory cytokines and DAMPs can further propagate and aggravate inflammation.

#### 2.3.2 GSDME-mediated pyroptosis

Additionally, under conditions of functional caspase-1 deficiency or prolonged/impaired stimulus timing (Antonopoulos et al., 2015; Gan et al., 2025), NLRP3 inflammasome activation can also alternatively initiate a proteolytic cascade of successive caspase-8, caspase-3, and GSDME cleavage (Chao et al., 2023). GSDME, another crucial member of the gasdermin family, has emerged as a molecular switch between apoptosis and pyroptosis. Upon its activation by caspase-3, GSDME undergoes cleavage to release the pore-forming N-terminal fragment, thereby converting non-inflammatory apoptosis into inflammatory pyroptotic cell death (Wang et al., 2017c).

#### 2.3.3 Caspase-8 links NLRP3 inflammasome activation to multiple cell death pathways

In the context of NLRP3 inflammasome activation, caspase-8 not only plays a crucial role in the alternative pathway, but also acts as a regulator. A study has revealed that caspase-8 and its adaptor proteins FADD are indispensable apical mediators of both canonical and noncanonical NLRP3 inflammasome transcriptional priming and activation (Gurung et al., 2014). Besides its catalytic activity, in TLR3-mediated NLRP3 inflammasome activation, caspase-8 functions as a non-enzymatic scaffold protein that facilitates the assembly of the TRIF–RIPK1–FADD–caspase-8 complex, and subsequently drives post-translational priming of NLRP3 (Kang et al., 2015).

Instead of being well known as a traditional extrinsic apoptosis initiator, caspase-8 is now considered a central signaling hub linking the NLRP3 inflammasome with apoptosis, pyroptosis, and necroptosis (Fritsch et al., 2019). Typically, the NLRP3/ASC complex preferentially recruits caspase-1 rather than caspase-8. However, under the condition of a delayed triggering signal (such as nigericin) or caspase-1 deficiency, the recruitment diverts to caspase-8, which switches pyroptosis to apoptosis and secondary necrosis (Gan et al., 2025). Moreover, under specific conditions, caspase-8 can directly cleave GSDMD or indirectly activate GSDME through caspase-3 as mentioned above, enabling the conversion of apoptosis into pyroptosis (Gram et al., 2019). In addition, caspase-8 also suppresses RIPK3-MLKL-dependent necroptosis and shifts cell fate toward apoptosis (Bertheloot et al., 2021).

#### 2.3.4 PANoptosis: integration of multiple cell death pathways

Traditionally, apoptosis, pyroptosis, and necroptosis were viewed as parallel, mutually exclusive pathways. However, the multifaceted nature of caspase-8 has shattered this conventional paradigm. It is this trinity of interconnected cellular demise, tethered by caspase-8, that defines the phenomenon of PANoptosis. PANoptosis is a form of innate immune-mediated, lytic, and inflammatory cell death driven by caspases and receptor-interacting protein kinases (RIPKs) through the assembly of PANoptosome complexes (Pandeya and Kanneganti, 2024). Downstream of this coordinated signaling network, the activation of respective executioners – caspase-3/7 for apoptosis, gasdermins (GSDMD/E) for pyroptosis, and phosphorylated MLKL for necroptosis – leads to distinct morphological outcomes (Pandeya and Kanneganti, 2024). NLRP3, functioning as a sensor molecule, regulates PANoptosis via two major mechanisms: it directly triggers pyroptosis and promotes the maturation of pro-inflammatory cytokines, while also participating in PANoptosome assembly to coordinate PANoptotic cell death (Jiang et al., 2025). This framework may help explain the concurrent activation of apoptotic, pyroptotic, and necroptotic signatures increasingly reported in AMD models and patient tissues (He et al., 2025; Wang et al., 2026).

To sum up, these findings suggest that NLRP3 activation can orchestrate multiple forms of inflammatory cell death beyond cytokine maturation alone. In the context of AMD, such mechanisms provide a framework for understanding how chronic retinal stress may translate into progressive degeneration of retinal cells. The following section therefore describes the evidence linking these pathways to AMD pathogenesis.

## 3 The role of the NLRP3 inflammasome in AMD

To understand how NLRP3 contributes to AMD progression, it is necessary to integrate AMD-specific danger signals, the retinal cell populations that respond to these stimuli, the downstream inflammatory and cell-death pathways they activate, and the resulting disease phenotypes. Accordingly, this section discusses NLRP3 signaling from four interconnected perspectives: cell-type specificity, retinal DAMPs, inflammatory amplification, and stage-specific disease outcomes.

The involvement of the NLRP3 inflammasome in AMD was first established by two independent studies published in 2012 (Doyle et al., 2012; Tarallo et al., 2012). In Doyle’s study, drusen isolated from AMD patients’ eyes could activate the NLRP3 inflammasome and enhance IL-1β production. Subsequent analysis identified specific components in drusen, including carboxyethylpyrrole (CEP) protein adducts that are uniquely generated from the oxidation of docosahexaenoic acid-containing lipids and are more abundant in serum and drusen from AMD donors than those from non-diseased donors. Based on these findings, a dry AMD-like mouse model was established by immunizing the mice with CEP-adducted mouse serum albumin. In these mice, NLRP3 and caspase-1 were discovered in activated macrophages in the retinas.

In a 2011 study, Alu RNA accumulation due to RNase DICER1 deficiency was observed in RPE cells from human eyes with GA (Kaneko et al., 2011). Subsequently, Tarallo et al. demonstrated that Alu RNA could induce RPE cell death by activating the NLRP3 inflammasome (Tarallo et al., 2012). Furthermore, a case report in 2022 described early-onset drusen formation and RPE dysfunction in a 38-year-old patient with an NLRP3-associated autoinflammatory disease (NLRP3-AID) (Li et al., 2023). Although no genetic risk studies to date have established a relationship between polymorphism of NLRP3 and AMD risk, all these lines of evidence link NLRP3 inflammasome activation to the mechanism and development of AMD (Brandli et al., 2024).

### 3.1 Cell-type-specific roles of the NLRP3 inflammasome across AMD progression

In the retina, various cell types, such as RPE, microglia, macrophages, and endothelial cells, can express NLRP3. Among these, RPE cells play a crucial role in maintaining normal retinal structure and function, including phagocytosis of photoreceptor outer segments (POS), forming the outer blood-retinal barrier (BRB), transportation of nutrients and ions, and supporting the visual cycle. RPE cells also have an immunomodulation effect by orchestrating both innate and adaptive immunity (Wang et al., 2023). Therefore, dysfunction of RPE cells may initiate AMD pathology. Under physiological conditions, RPE cells only express NLRP3, IL-18, and IL-1β at low levels. However, sustained chronic activation of the NLRP3 inflammasome is induced during aging when RPE cells are exposed to oxidative stress, lysosomal destabilization, and other inflammatory stimuli (Kauppinen et al., 2012; Tseng et al., 2013). As AMD progresses to the late stage, RPE cells inevitably undergo cell death, which further exacerbates oxidative stress and inflammation (Zhang et al., 2025). Therefore, RPE cells may represent both a primary source and a major target of NLRP3-mediated inflammation, placing them at the center of AMD pathogenesis.

Retinal microglia are the resident macrophages of the retina, whose main function is to remove cellular debris and aged synapses. In the pathogenesis of AMD, microglia are activated in response to the primary death of retinal neurons and migrate toward the ongoing retinal lesions. Activated microglia can release pro-inflammatory cytokines, including IL-1β and IL-18, through NLRP3 inflammasome activation, which amplifies inflammatory responses, RPE degeneration, and AMD progression (Zhao et al., 2024). Knockout of C–C motif chemokine receptor-2 (Ccr2) inhibited the microglial migration in the retina and suppressed the activation of NLRP3 inflammasome within microglia, thereby alleviating chronic blue light-induced retinopathy (Hu et al., 2016). Furthermore, NLRP3 inflammasome activation in the activated macrophages and retinal microglia, rather than in RPE cells, promotes VEGF-A-induced choroidal neovascularization (CNV) through the secretion of IL-1β (Marneros, 2023). Notably, these findings suggest that microglial NLRP3 activation is not merely a downstream consequence of retinal injury but may function as an active driver that links initial retinal damage to sustained inflammation and subsequent pathological neovascularization during AMD progression.

Müller glial cells are the main macroglia of the retina that provide basic structural and functional support for retinal homeostasis (Newman and Reichenbach, 1996). In early AMD, Müller cells act in a compensatory manner to preserve retinal homeostasis under stress. As the disease advances, they shift toward maladaptive remodeling and contribute to structural disintegration. In late AMD, Müller cells form glial seals and enter senescence-like states, marking a transition from neuroprotection to containment and degeneration (Chen et al., 2026). Increasing evidence suggests that activated Müller cells also participate in retinal neuroinflammation. *In vitro*, chemokine expression in Müller glial cells is upregulated when stimulated with microglia-derived IL-1β (Natoli et al., 2017). However, the precise relationship between Müller cell reactivity and NLRP3 activation remains incompletely understood. Importantly, NLRP3 deletion protected against BLamD formation but did not suppress Müller cell gliosis in the R345W Efemp1 mutation model, indicating that Müller cell reactivity is NLRP3-independent (Ortega et al., 2025).

Choriocapillaris is a single fenestrated capillary layer that supplies oxygen and nutrients to the RPE and photoreceptors, as well as removes waste. Choroidal endothelial cell degeneration is considered an early pathogenic event in AMD and may precede RPE dysfunction (Chirco et al., 2017). Accumulation of membrane attack complex in aging choriocapillaris may lead to cell lysis and upregulation of proangiogenic factors in surviving cells, which is a major trigger for AMD progression (Zeng et al., 2016). Hypoxic RPE cells secreted ANXA1 that inhibited NLRP3 inflammasome activation and subsequent pyroptosis in CECs via the FPR2/SHP2 pathway. Moreover, ANXA1 promoted proliferation, migration, and tube formation of the surviving CECs, thereby leading to CNV formation (Zhu et al., 2022). This finding highlights the complexity of inflammasome signaling in neovascular AMD, as suppression of NLRP3-mediated pyroptosis in CECs may inadvertently enhance endothelial survival and angiogenic activity, thus promoting CNV formation.

These data reveal that NLRP3 activation is highly cell-type dependent within the retinal microenvironment. While activation in RPE cells and microglia predominantly promotes degeneration and inflammation, its role in Müller cells remains incompletely defined, and endothelial inflammasome signaling may exert paradoxical effects on angiogenesis.

### 3.2 Retinal DAMPs and microenvironmental triggers of NLRP3 activation

**FIGURE 2:**
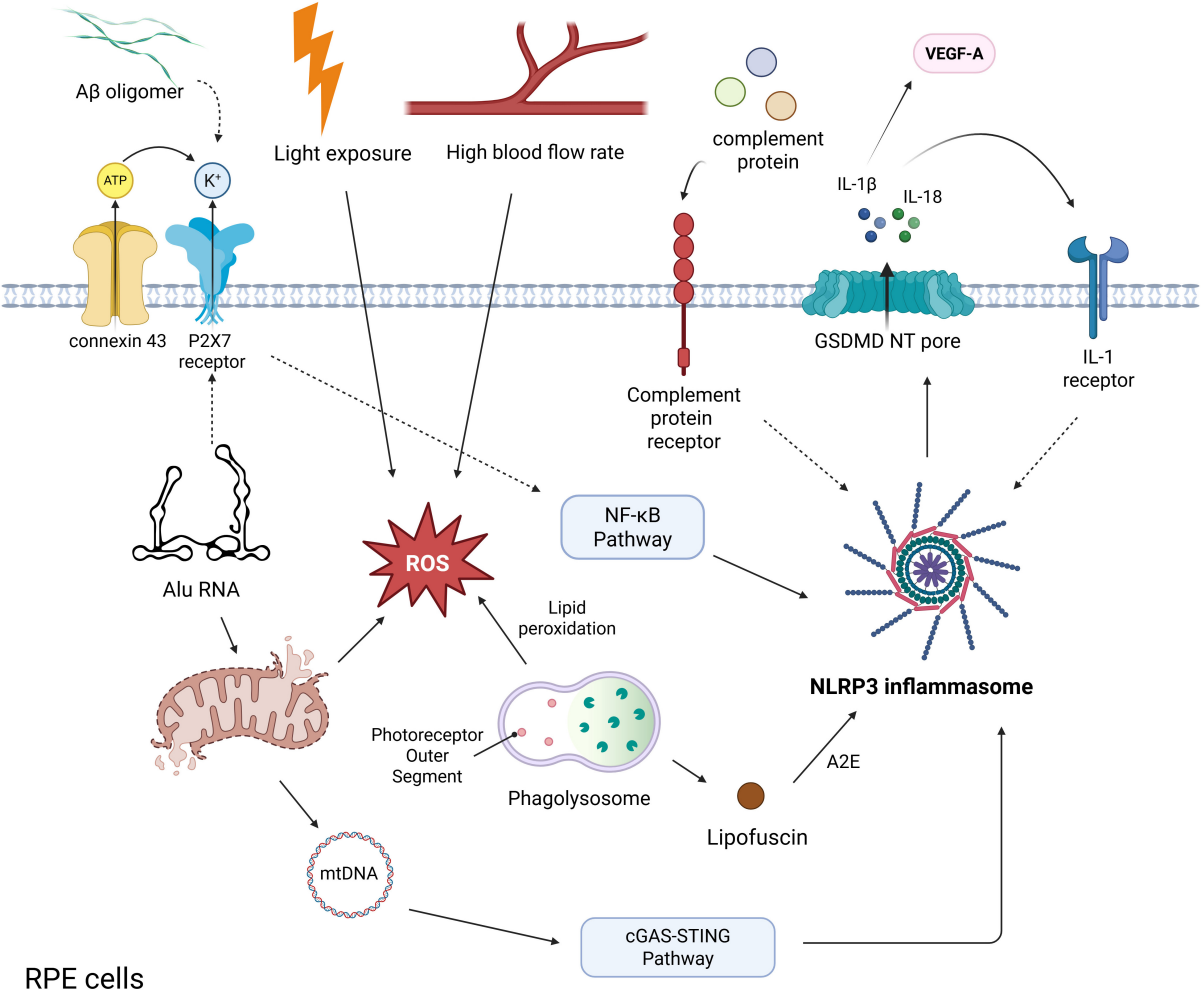
Mechanism of NLRP3 inflammasome activated by DAMPs in the retina. The retina harbors several DAMPs that either originate from stressed cells or are present within drusen. The retina provides an ideal condition for the generation of ROS, because of the cumulative light exposure, high blood flow rate, and the oxidation of polyunsaturated fatty acids existing in the photoreceptor membranes. Extracellular ATP is elevated under stressed conditions, and high concentrations of ATP sensed by the P2X7 receptor trigger ionic fluxes that subsequently activate the NLRP3 inflammasome. P2X7 is also required for NLRP3 inflammasome activation induced by Aβ and *Alu* RNA. Additionally, *Alu* RNA can mediate mitochondria disruption, releasing mtDNA that in turn activates the non-canonical pathway of NLRP3 inflammasome via the cGAS-STING pathway. Lipofuscin in RPE cells is mostly derived from the phagocytosis of POS. The major component, A2E, can activate the NLRP3 inflammasome directly. IL-18 and IL-1β released by GSDMD in RPE cells can bind to their corresponding receptors to propagate inflammation. IL-1β is also known as a pro-angiogenic factor that can stimulate the secretion of VEGF-A in human RPE cells and thus promote wet AMD. The dashed lines indicate that the effector may not be triggered directly. This figure was created in https://BioRender.com.

The retina is continuously exposed to diverse stimuli originating from stressed cells or drusen components. Common danger signals include ROS, lysosomal damage, and ionic flux triggered by extracellular ATP. Additionally, drusen constituents such as Aβ oligomer and complement proteins, as well as *Alu* RNA and lipofuscin that accumulate in RPE cells, are also DAMPs that potently activate the NLRP3 inflammasome (Figure 2).

#### 3.2.1 Reactive oxygen species (ROS) and oxidative stress

As previously discussed, ROS serves as a key initiator of NLRP3 inflammasome assembly (Abais et al., 2015). Elevated ROS levels that cause cellular damage have been strongly implicated in several diseases, particularly age-related disorders (Beatty et al., 2000). The retina provides an ideal condition for the generation of ROS. First, the retina is continuously exposed to cumulative photo-oxidative stress from light irradiation. Second, the RPE cells exhibit a high metabolic demand, which necessitates elevated oxygen consumption supported by high choroidal blood flow and enriched mitochondria. Third, in the RPE cells, during the phagocytosis of POS, the abundance of polyunsaturated fatty acids in the photoreceptor outer segment membranes is prone to lipid peroxidation that can initiate a cytotoxic chain reaction (Datta et al., 2017; Winkler et al., 1999). Thus, the macular region, particularly RPE cells, resides in a chronically oxidative microenvironment that favors persistent ROS accumulation and consequently provides a permissive setting for sustained NLRP3 inflammasome activation.

#### 3.2.2 Drusen-associated components

**Complement proteins**

Complement proteins are among the major inflammatory components of drusen. Drusen component C1Q, the primary initiating component in the complement pathway, can activate the NLRP3 inflammasome and elevate IL-18 and IL-1β levels via altering phagolysosome activity and inducing cathepsin B leakage (Doyle et al., 2012). In RPE cells, normal human serum was found to prime the inflammasome that was further activated by lipofuscin-mediated photo-oxidative damage. This priming effect could be inhibited by C5 deletion or C5a receptor blockade, indicating C5 as an active agent (Brandstetter et al., 2015a). Another RPE cell culture model showed that NLRP3 expression was up-regulated under hyperosmotic stress, hypoxia, and oxidative stress, which was suppressed by C9 siRNA-mediated gene knockdown, suggesting that C9 might contribute to NLRP3 inflammasome activation (Hollborn et al., 2018). Furthermore, complement factor H-related-3 (FHR-3), locally expressed in eyes by retinal macrophages and microglia, can be internalized by RPE cells. This internalization upregulates the expression of the complement and the complement receptors, and thereby primes the NLRP3 inflammasome (Schäfer et al., 2021). In sum, these findings indicate that complement activation contributes to both the priming and activation of the NLRP3 inflammasome, which may provide a mechanistic bridge between drusen accumulation and chronic retinal inflammation in AMD.

**Aβ oligomers**

Accumulation of amyloid-β (Aβ) occurs not only in the brain, but also in the retina. Of note, Aβ_1–40_ is a constituent of drusen in AMD (Lashkari et al., 2020). Intravitreal injections of Aβ_1–40_ in animal models significantly upregulated the expression of NLRP3, caspase-1, IL-18, and IL-1β, indicating that Aβ_1–40_ might be responsible for NLRP3 inflammasome activation in AMD pathogenesis (Liu et al., 2013). It has also been shown that Aβ can induce LPS-primed NLRP3 inflammasome activation in ARPE-19 cells via NADPH oxidase- and mitochondria-dependent ROS production, ultimately leading to RPE degeneration (Wang et al., 2017a). Moreover, the P2X7 receptor is also identified as a critical mediator for Aβ-induced RPE degeneration (Narendran et al., 2021). These observations indicate that Aβ can engage multiple upstream signals, including ROS production and P2X7 receptor activation, which ultimately drive the NLRP3 inflammasome signaling and promote RPE degeneration. The ability of Aβ to activate NLRP3 inflammasome signaling further supports the concept that AMD shares common inflammatory mechanisms with other age-related neurodegenerative disorders characterized by protein aggregation.

#### 3.2.3 Lipofuscin and A2E

Lipofuscin is an auto-fluorescent material that accumulates over time in lysosomes of various cell types throughout the body (Fleckenstein et al., 2021). Previously, the presence of lipofuscin within RPE cells was considered to be the earliest indicator of senescence in the outer retinal layer (Young, 1988). Unlike other cell types in which lipofuscin arises primarily from the autophagic degradation of intracellular organelles, lipofuscin in RPE cells is predominantly derived from the phagocytosis of POS (Datta et al., 2017). In RPE cells, photo-oxidative stress induced by blue light irradiation can activate the inflammasome via lysosomal destabilization, which can be amplified by the accumulation of lipofuscin (Brandstetter et al., 2015b). However, lipofuscin accumulation alone did not fully account for this effect. Instead, the bisretinoid compound *N*-retinyl-*N*-retinylidene ethanolamine (A2E), a major component of lipofuscin and a byproduct of the visual cycle, was competent to directly activate the NLRP3 inflammasome (Anderson et al., 2013; Sparrow et al., 2003). In addition, A2E disturbs the regulatory function of RPE cells in suppressing Th1 through the secretion of IL-1β and inhibition of prostaglandin E2 (PGE2), which indicates that A2E might contribute to the immune pathogenesis of AMD (Shi et al., 2015).

#### 3.2.4 Nucleic acid stress and innate immune sensing

*Alu* RNA is a double-stranded RNA encoded by small repetitive *Alu* elements in the human genome, and its high-level expression has been linked to numerous human diseases (Deininger, 2011). As previously mentioned, *Alu* RNA accumulation occurs in RPE cells of GA samples due to DICER1 deficiency. Mechanistically, *Alu* RNA can prime the NLRP3 inflammasome through the NF-κB pathway in a P2X7 receptor-dependent manner, independently of TLR signaling (Kerur et al., 2013). Furthermore, cyclic GMP–AMP synthase (cGAS), a cytosolic DNA sensor, plays a critical role in mediating *Alu*-induced RPE cell death (Li et al., 2022). In RPE cells, *Alu* RNA accumulation or DICER1 knockdown results in mitochondria disruption and the release of mtDNA into the cytoplasm through the mitochondrial permeability transition pore (mPTP). The cGAS engaged by mtDNA then activates the noncanonical pathway of the NLRP3 inflammasome via the cGAS-STING signaling pathway (Kerur et al., 2018).

#### 3.2.5 ATP signaling and purinergic pathways

Under cellular stress, intracellular nucleotides like ATP and UTP that function as danger signals are released into the extracellular space (Gallucci and Matzinger, 2001). The ATP release is primarily mediated by connexin 43 hemichannels that open in response to cell injury. These hemichannels are widely expressed in retinal cells such as microglia, Müller cells, and RPE cells and are responsible for the propagation of inflammation (Danesh-Meyer et al., 2016; Mugisho et al., 2018). High concentrations of extracellular ATP can activate the P2X7 receptor, a subtype of purinergic receptors. The activation subsequently triggers K^+^ efflux and Na^+^ influx that drive NLRP3 inflammasome assembly (Di Virgilio et al., 2017).

#### 3.2.6 Nuclear proteins

High mobility group box (HMGB) proteins are DNA-binding proteins that critically regulate genomic maintenance processes, including DNA replication, transcription, and repair. Under cellular stress conditions, these nuclear proteins are translocated into the cytosol and released during membrane rupture. The extracellular HMGB family proteins, notably HMGB1, are regarded as DAMPs that can initiate the NLRP3 inflammasome signaling pathway and subsequent inflammatory response (Jia et al., 2019; Ding et al., 2023). A2E-induced upregulation of HMGB1 and caveolin-1, coupled with subsequent HMGB1 release, may relate to RPE cell senescence and the pathogenesis of AMD (Sun et al., 2019). Moreover, another HMGB family protein member, HMGB2, has also been proven to mediate photoreceptor death under oxidative stress via upregulating NF-κB/NLRP3 and simultaneously downregulating Nrf2/HO-1 signaling pathways. Knockdown of HMGB2 could rescue retina degeneration caused by photo-oxidative stress, which proposed HMGB2 as a therapeutic target of AMD (Zhang et al., 2022).

Altogether, these diverse retinal DAMPs cooperatively drive NLRP3 inflammasome signaling despite their distinct origins and mechanisms of action. This central role places NLRP3 at the hub of the AMD inflammatory network, where persistent activation disrupts retinal homeostasis and contributes to disease progression across different AMD stages and phenotypes.

### 3.3 Crosstalk between NLRP3 inflammasome and SASP in AMD

Senescence-associated secretory phenotype (SASP) is defined as the phenomenon in which senescent cells secrete a broad range of bioactive molecules, including cytokines, chemokines, growth factors, and other factors (Wang et al., 2024). Given that AMD is an aging disease, SASP is increasingly recognized as an important contributor to its pathogenesis, for it seems that SASP may partly explain the low-level chronic inflammation within tissues (Herranz and Gil, 2018; Blasiak, 2020). In response to various stressors, including oxidative stress, DNA damage, activation of cGAS-STING, and Aβ accumulation, senescent retinal cells, especially RPE cells, develop a SASP and subsequently release numerous inflammatory and angiogenic mediators such as IL-1β, IL-6, IL-8, VEGF, and matrix metalloproteinase-9 (MMP-9). These SASP factors can propagate inflammation, disrupt BRB, promote CNV, and remodel the subretinal microenvironment, consequently contributing to AMD progression (Blasiak, 2020).

The bidirectional crosstalk between NLRP3 inflammasome activation and SASP represents a critical feed-forward loop driving retinal degeneration. On the one hand, SASP-derived factors can act as DAMPs that provide priming and activation signals required for NLRP3 inflammasome assembly. On the other hand, SASP cytokine IL-1β is produced via the canonical NLRP3 inflammasome pathway. Together, these reciprocal interactions establish a self-perpetuating inflammatory cycle that reinforces cellular senescence, amplifies chronic sterile inflammation, and accelerates retinal aging and AMD progression (Acosta et al., 2013; Wang et al., 2024; Blasiak, 2020).

### 3.4 NLRP3-associated inflammatory cell death in AMD

Pyroptosis mediated by the NLRP3 inflammasome is one of the major cell death mechanisms in AMD progression (Sun et al., 2024). Upon activation, the NLRP3 inflammasome triggers caspase-1-mediated cleavage of gasdermin D (GSDMD) and maturation of IL-18 and IL-1β. A photo-oxidative damage murine model showed that caspase-1-dependent inflammasomes were responsible for the propagation of inflammation and cell death during retinal degeneration. An increased level of photoreceptor cell death was positively correlated with upregulation of caspase-1. However, the sole knockout of caspase-11 exerted no protective role against photo-oxidative damage (Wooff et al., 2020).

Gasdermin D, a key executor of pyroptosis, is also involved in retinal degeneration. A mutation (GSDMD^*I105N*/*I105N*^) that impairs the pore-forming activity has been shown to preserve retinal function by inhibiting cytokine release and preventing cell death. However, genetic knockout of GSDMD did not yield a similar protective effect. This discrepancy may be attributed to other pore-forming proteins like GSDME that could compensate for the absence of GSDMD. Moreover, the study also suggested that a portion of IL-1β may be secreted via encapsulation within extracellular vesicles (EVs) (Sekar et al., 2023).

IL-18 and IL-1β are members of the IL-1 cytokine family. As discussed above, after maturation, IL-18 and IL-1β are released through pores formed by GSDMD. IL-1β is known as a pro-angiogenic factor that can promote the secretion of VEGF-A and VEGF-C in human RPE cells (Nagineni et al., 2012), so it is often associated with the pathogenesis of neovascular AMD. IL-1β secreted by microglia also has the ability to stimulate Müller cells and RPE to express chemokines such as Ccl2, Cxcl1, and Cxcl10, thereby exacerbating retinal degeneration (Natoli et al., 2017). In addition, the tight junctions of RPE cells can also be altered by IL-1β, which increases the permeability of the outer BRB and may result in the leakage of molecules and cells from the circulatory system to the ocular environment (Abe et al., 2003).

Recently, IL-18 has been proposed to be a potential plasma inflammatory biomarker of the intermediate stage AMD in individuals with AIDS (Jabs et al., 2023). Beyond its potential value as a biomarker, IL-18 has also been implicated in AMD pathogenesis. IL-18 is recognized as an effector of *Alu* RNA-induced RPE cell death, for IL-18 neutralization, but not IL-1β, reversed it (Tarallo et al., 2012). However, the role of IL-18 in neovascular AMD remains controversial. While Doyle et al. reported that IL-18 exerted anti-angiogenic effects and suppressed CNV, Hirano et al. failed to confirm this protective effect and raised concerns regarding potential retinal toxicity associated with exogenous IL-18 administration (Doyle et al., 2014; Hirano et al., 2014).

Besides the classical caspase-1/GSDMD axis, emerging evidence suggests that other additional NLRP3-associated cell death pathways may also operate in the retinal microenvironment. GSDME has been recognized as the key executor of all-trans-retinal (atRAL)-induced photoreceptor damage in retinal degeneration associated with AMD. During photoreceptor loss, GSDME can not only participate in pyroptosis but also reinforce apoptosis by stimulating upstream activation of caspase-3 (Cai et al., 2022). Moreover, crocin has been demonstrated to reduce the cleavage of GSDME by repressing the activity of caspase-3, thereby resisting the photoreceptor pyroptosis induced by atRAL (Yang et al., 2024).

Recent studies in AMD have increasingly focused on PANoptosis. He et al. have revealed that Aβ_1–40_ can induce PANoptosis-like cell death both *in vivo* and *in vitro*, as evidenced by the simultaneous activation of apoptotic, pyroptotic, and necroptotic pathways, along with the upregulation of AIM2-PANoptosome-associated components, including AIM2, ASC, and NLRP3 (He et al., 2025). Furthermore, a more recent study demonstrated that oligomerization of VDAC1, a membrane protein on the mitochondria, promoted mitochondrial DNA release and activated the mtDNA-STING signaling axis, hence triggering subsequent PANoptosis and aggravating RPE degeneration in AMD (Wang et al., 2026). In conclusion, these findings highlight PANoptosis as a unified cell death pathway in AMD pathogenesis, offering fresh mechanistic insights into retinal degeneration.

### 3.5 Stage-specific roles of NLRP3 across AMD progression

Taken together, current evidence indicates that NLRP3 inflammasome activation exerts distinct biological effects across AMD progression. During early and intermediate AMD, NLRP3 primarily acts as an amplifier of chronic sterile inflammation in response to drusen accumulation, oxidative stress, complement activation, and cellular senescence (Doyle et al., 2012; Kauppinen et al., 2012; Brandstetter et al., 2015a,Wang et al., 2024), driving progressive but subclinical disruption of retinal homeostasis prior to obvious cell loss. As the disease progresses toward GA, persistent inflammasome activation promotes severe RPE dysfunction, GSDMD-mediated pyroptosis, and photoreceptor degeneration, thereby contributing directly to irreversible retinal atrophy (Wooff et al., 2020; Sekar et al., 2023). In contrast, in nAMD, NLRP3 appears to exert a more complex role, with inflammasome-derived cytokines such as IL-1β facilitating angiogenic signaling and CNV formation (Nagineni et al., 2012), whereas some downstream mediators such as IL-18 may display context-dependent or even opposing regulatory effects on choroidal neovascularization (Hirano et al., 2014; Doyle et al., 2012).

These observations suggest that the pathological consequences of NLRP3 activation are highly dependent on the specific disease stage, cellular context, and downstream effector pathways – a multifaceted nature that partly explains the mixed outcomes observed with broad NLRP3 inhibition in experimental AMD models. For clarity, the stage-specific roles of NLRP3 across AMD progression are summarized in Table 1.

**TABLE 1:** Stage-specific roles of the NLRP3 inflammasome across AMD progression.

AMD stage/phenotype	Major activating stimuli	Predominant cell types	Key downstream mediators	Major consequences of NLRP3 activation	Representative evidence
Early/intermediate AMD	Drusen components, complement activation, oxidative stress, Aβ, lipofuscin	RPE, microglia	IL-1β, IL-18	Chronic para-inflammation, inflammasome priming, RPE dysfunction, amplification of retinal inflammation	Doyle et al., 2012; Kauppinen et al., 2012; Liu et al., 2013
Geographic atrophy (GA)	*Alu* RNA accumulation, DICER1 deficiency, mtDNA release, persistent oxidative stress	RPE, microglia	GSDMD, IL-18	Pyroptosis, PANoptosis, RPE degeneration, photoreceptor loss	Tarallo et al., 2012; Kerur et al., 2018; Wooff et al., 2020; Sekar et al., 2023
Neovascular AMD (nAMD)	Hypoxia, cytokine signaling, macrophage/microglial activation	Microglia, macrophages, RPE, choroidal endothelial cells	IL-1β, VEGF	IL-1β secretion, angiogenic signaling, CNV formation, vascular remodeling	Marneros, 2023; Natoli et al., 2017; Zhu et al., 2022

## 4 Future perspective on therapies targeting the NLRP3 inflammasome signaling pathway

To date, a wide range of therapeutic candidates have been proposed to target the NLRP3 inflammasome pathway in AMD. However, despite encouraging preclinical findings, none has yet advanced to clinical application specifically for AMD. Moreover, their translational readiness varies considerably. To facilitate comparison, the candidates summarized in Table 2 are additionally stratified according to their targets and translational potential, ranging from clinically approved drugs with repurposing potential to early-stage experimental approaches. The following sections discuss these strategies according to their mechanisms of action while also considering the challenges associated with their clinical translation.

**TABLE 2:** Potential therapeutic strategies targeting the NLRP3 inflammasome and associated pathways in AMD.

Drug class	Representative agent	Model	Main findings	Translational stage*	References
Inflammasome-targeting therapies	IFM-632 and IFM-514	pRPE cell and ARPE-19 cell models	Suppressed inflammasome activation and IL-1β by direct NLRP3 inhibition	C	Wang et al., 2019
	TatCARD	*LXRα^–/–^* mice dry AMD-like model	Preserved RPE function and reduced IL-1β by interrupting the recruitment of procaspase-1	C	Choudhary et al., 2022
	Fluoxetine	*Alu* RNA-induced RPE degeneration models (*in vitro* and *in vivo*)	Prevented RPE degeneration by directly inhibiting both NLRP3-ASC inflammasome assembly and activation	A	Ambati et al., 2021
	Tranilast	Auranofin-treated ARPE-19 cell model	Inhibited NLRP3 inflammasome activation by enhancing NLRP3 ubiquitination	A	Yumnamcha et al., 2019
Upstream signaling inhibitors of NLRP3 activation	Vinpocetine	Aβ-induced retinal degeneration models (*in vitro* and *in vivo*)	Downregulated NF-κB signaling and NLRP3 activation	A	Liu et al., 2012
	TatM013	ARPE-19 cell model	Reduced IL-1β secretion by inhibiting NF-κB and inflammasome activation	C	Ildefonso et al., 2015
	Cyanidin-3-glucoside	4-hydroxyhexenal-treated ARPE-19 cell model	Protected RPE from inflammatory damage by suppressing the JNK-c-Jun/AP-1 pathway	B	Jin et al., 2018
	Amlexanox	Auranofin-treated ARPE-19 cell model	Attenuated retinal inflammation by suppressing TBK1/IKKε signalings	A	Yumnamcha et al., 2019
P2X7 inhibitors	NRTIs	Laser-induced CNV	Suppressed laser-induced CNV activation and downregulated VEGF-A	A	Mizutani et al., 2015
	Kamuvudines	Aβ_1–40_-treated dry AMD mice model	Both inhibited AβOs-induced RPE degeneration	A	Narendran et al., 2021
	P2X7 antagonist A740003	ox-LDL-treated models (*in vitro* and *in vivo*)	Reduced retinal inflammation and neovascularization	C	Yang et al., 2021
Connexin 43 inhibitors	Tonabersat	Light Damage Model of dry AMD	Preserved retinal photoreceptor function by blocking connexin 43	C	Mat Nor et al., 2020
	Peptide 5	Light Damage Model of dry AMD	Improved functional outcomes of neurons by blocking connexin 43	C	Guo et al., 2016
miRNA-based therapies	MicroRNA-22-3p	LPS and rotenone-treated ARPE-19 cell model & Blue light exposure mice model	Suppressed RPE damage by directly binding NLRP3 mRNA	C	Hu et al., 2019
	MicroRNA-191-5p	Aβ-induced retinal degeneration models (*in vitro* and *in vivo*)	Alleviated RPE cell injury by repressing C/EBPβ (a transcription factor of NLRP3)	C	Chen et al., 2021
	MicroRNA-21 inhibitor	Blue light exposure mice model	Inhibited NLRP3 inflammasome activation by reducing miR-21 expression	C	Zhang and Xu, 2025
	Baicalin	Aβ_1–40_-treated ARPE-19 cell model	Inhibited pyroptosis by upregulating miR-223	B	Sun et al., 2020
Cellular stress modulators	Puerarin	Aβ_1–40_-treated ARPE-19 cell model	Inhibited NLRP3 inflammasome activation by activating Nrf2/HO-1 antioxidant signaling pathway	B	Wang et al., 2017b
	SS31	Auranofin-treated ARPE-19 cell model	Restored mitochondrial function by scavenging mitochondrial ROS	C	Yumnamcha et al., 2019
	Resvega	IL-1α+MG-132 + BafA-treated ARPE-19 cell model	Alleviated NLRP3 inflammasome-mediated inflammation by reducing ROS and inducing autophagy	B	Bhattarai et al., 2021
	Epigallocatechin gallate (EGCG) and Resveratrol (RSVL)	*Alu* RNA-induced RPE degeneration ARPE-19 cell model	Protected RPE from cell death by inhibiting the cGAS pathway	B	Li et al., 2022
	Melatonin	Sodium iodate (SI)-induced retinal degeneration models (*in vitro* and *in vivo*)	Protected RPE cells from NLRP3 activation by promoting Ca^2+^ homeostasis	A	Ren et al., 2024
Hsp 90 inhibitors	Geldanamycin (has ocular toxicity)	IL-1α+MG-132 + BafA-treated ARPE-19 cell model	Promoted NLRP3 degradation by inhibiting Hsp 90	C	Piippo et al., 2018
	TAS-116	IL-1α+MG-132 + BafA-treated ARPE-19 cell model	Promoted NLRP3 degradation by inhibiting Hsp 90	C	Ranta-Aho et al., 2021
Cytokine signaling modulators	Rytvela	Blue light exposure mice model	Blocked pathological IL-1β signaling by IL-1R modulation	C	Dabouz et al., 2020

A*. Clinically approved drugs with preclinical AMD evidence; B. Clinically available nutraceuticals / supplements with preclinical AMD evidence; C. Experimental therapeutics.

### 4.1 Direct targeting of the NLRP3 inflammasome pathway

**FIGURE 3:**
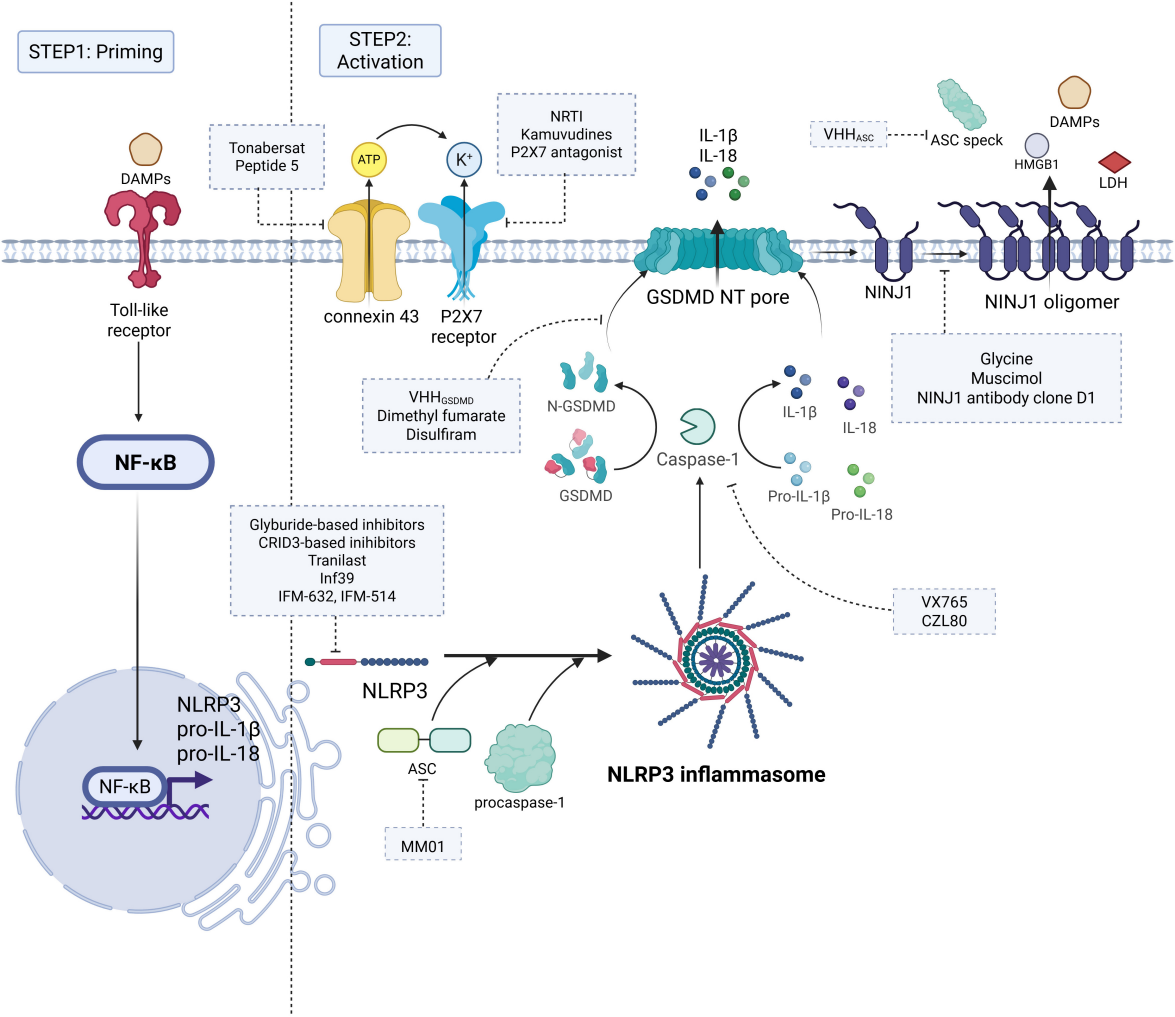
Inhibitors and antibodies targeting key checkpoints in the NLRP3 inflammasome pathway. NLRP3 inhibitors are primarily classified into two categories: glyburide-based inhibitors and CRID3-based inhibitors. CRID3, also known as MCC950, is a widely used selective inhibitor of NLRP3. Due to the small size and the ability to cross physiological barriers, VHH antibodies have been employed to target extracellular ASC specks and cytosolic GSDMD-NT. Glycine, muscimol, and NINJ1 neutralizing antibody clone D1 have been shown to prevent NINJ1 clustering, suppress membrane rupture, and consequently confer cytoprotection. This figure was created in https://BioRender.com.

There are a variety of drugs that directly target the NLRP3 inflammasome pathway, such as NLRP3, ASC, and GSDMD inhibitors or antibodies. Some of the commonly used agents are listed in Figure 3. NLRP3 inhibitors are primarily classified into two categories: glyburide-based inhibitors and cytokine release inhibitory drug 3 (CRID3)-based inhibitors, both of which are sulfonylurea derivatives. CRID3, also known as MCC950, is a widely used selective inhibitor of NLRP3 (Coll et al., 2015). Cryo-electron microscopy studies of full-length NLRP3 bound to MCC950 suggest that CRID3 exerts its inhibitory effect by stabilizing the inactive conformation of NLRP3 (Hochheiser et al., 2022). However, although MCC950 has demonstrated efficacy in various preclinical inflammatory disease models, its application in AMD remains limited and complicated. Paradoxically, NLRP3 inhibition by MCC950 increased CNV lesion size in a laser-induced choroidal neovascularization model, suggesting that indiscriminate NLRP3 blockade may not be beneficial and warrants caution in the context of AMD (Dieckmann et al., 2024). This evidence indicates that the role of NLRP3 may vary across different AMD phenotypes and disease stages, emphasizing the need for cell-type- and stage-specific therapeutic strategies.

Beyond experimental NLRP3 inhibitors, drug repurposing has emerged as an attractive translational strategy. Fluoxetine, a selective serotonin reuptake inhibitor (SSRI) that is widely used for the treatment of depression, has been recently identified as a direct NLRP3 inhibitor capable of binding the NACHT domain and preventing inflammasome assembly (Ambati et al., 2021). In experimental models of atrophic AMD, fluoxetine reduced RPE degeneration and inflammasome activation, supporting its potential therapeutic value within the degenerating retina. Importantly, unlike newly developed inflammasome inhibitors, fluoxetine benefits from an established clinical safety profile and extensive human use, which may substantially accelerate clinical translation. Nevertheless, whether the retinal protective effects observed in preclinical studies are primarily attributable to NLRP3 inhibition or involve additional pharmacological actions remains to be clarified.

Nanobodies, also known as VHH antibodies, refer to a unique form of immunoglobulin G originally found in *Camelidae*. Unlike conventional antibodies, VHH antibodies are devoid of the light chain polypeptide and the first constant domain (CH1), resulting in a smaller size that allows them to reach cryptic epitopes inaccessible to bulkier antibodies. Notably, four hydrophobic amino acids in the framework region 2 (FR2) are substituted with hydrophilic residues, which enhances their ability to cross physiological barriers (Muyldermans, 2013). Leveraging these advantages, VHH antibodies have been employed to target extracellular ASC specks and cytosolic GSDMD-NT in non-ocular inflammasome models, and thus can inhibit the propagation of inflammasome-mediated pathology and block the pore formation on the membrane, respectively (Bertheloot et al., 2022; Schiffelers et al., 2024). Although their efficacy in AMD has not yet been evaluated, their small size and favorable tissue-penetrating properties may be advantageous for retinal delivery and precise targeting of inflammasome components.

Regulation of proteins and their mRNAs through various modifications represents another feasible therapeutic strategy. In the case of proteins, post-translational modifications (PTMs), referring to the covalent attachment or removal of specific chemical groups to/from the amino acid residues, can regulate the priming and assembly processes of inflammasomes. For instance, the anti-inflammatory drug Tranilast promotes NLRP3 ubiquitination at the NACHT domain and subsequently disrupts inflammasome assembly (Huang et al., 2018). A study has shown that a combination of SS31 (mitochondrial antioxidants), amlexanox (another anti-inflammatory drug), and tranilast can preserve the RPE cell viability impaired by auranofin, an inhibitor of the Trx/TrxR system (an antioxidant system) (Yumnamcha et al., 2019).

Regarding the regulation of mRNAs, microRNAs (miRNAs) are endogenous non-coding single-stranded RNAs with a length of approximately 22 nt. The miRNAs can bind to the complementary sequences on target mRNAs, and thus slice the mRNAs and prevent their translation. For example, overexpression of microRNA-22-3p remarkably reduces RNA and protein expression of NLRP3, caspase-1, and IL-1β, thus protecting the primed RPE cells (Hu et al., 2019). In contrast, while microRNA-22-3p suppresses the NLRP3 inflammasome, microRNA-21 is associated with the upregulation of NLRP3. It has been shown that the microRNA-21 inhibitor can alleviate the cell damage caused by photo-oxidative stress (Zhang and Xu, 2025).

Heat shock protein 90 (Hsp90) is a multifunctional molecular chaperone that protects NLRP3 from degradation. Upon secondary signal stimulation, Hsp90 releases NLRP3, allowing its oligomerization and following inflammasome assembly. Inhibition of Hsp90 by geldanamycin leads to NLRP3 degradation by autophagy, as evidenced by the fact that this effect is diminished by the autophagy inhibitor bafilomycin A (BafA) (Piippo et al., 2018). However, the clinical application of geldanamycin is not practical due to its ocular toxicity. Instead, TAS-116, a well-tolerated Hsp90 inhibitor, has exhibited a higher therapeutic index than geldanamycin (Ranta-Aho et al., 2021).

Overall, these studies demonstrate that direct targeting of inflammasome components or their regulatory machinery may provide greater mechanistic specificity than broad anti-inflammatory approaches. Nevertheless, most of these strategies remain at the preclinical stage, and their efficacy, delivery feasibility, and long-term safety in AMD require further validation.

### 4.2 Indirect modulation of upstream pathways associated with NLRP3 activation

In addition to direct inflammasome inhibition, several interventions have been reported to attenuate NLRP3 activation indirectly by modulating oxidative stress, NF-κB signaling, calcium homeostasis, or autophagy. However, because these canonical pathways are ubiquitously active across multiple organ systems, most of these approaches remain exploratory and are not specific to AMD or the NLRP3 inflammasome itself. Therefore, their therapeutic value should be interpreted in the context of AMD-specific pathogenic mechanisms rather than generalized anti-inflammatory effects.

The activation of the NF-κB pathway is essential for NLRP3 priming. Vinpocetine, a selective inhibitor of phosphodiesterase 1 (PDE1), is able to suppress Aβ-induced NF-κB activation both *in vitro* and *in vivo*, and thus downregulates the expression of NLRP3 and related genes (Liu et al., 2012). This holds relevance for AMD, as Aβ peptide accumulation is a well-documented constituent of sub-RPE drusen deposits. Similarly, AAV-delivered TatM013, an NF-κB inhibitor, significantly reduces the secretion of IL-1β in the ARPE-19 cell line by inhibiting NLRP3 inflammasome (Ildefonso et al., 2015).

Given that cumulative photo-oxidative stress and high metabolic demands drive NLRP3 inflammasome activation in the outer retina, targeting this pathway with antioxidants or modulating antioxidative signaling represents a promising therapeutic strategy. Sodium iodate (SI) is a well-established chemical agent that induces cytotoxic oxidative stress and activates the NLRP3 inflammasome in models of dry AMD. In a recent study utilizing the SI-induced RPE degeneration model, melatonin, an endogenous antioxidant, has been demonstrated to enhance cell viability and protect the oxidatively damaged retina by downregulating the expression of NLRP3 protein, caspase-1, IL-18, and IL-1β. This protective effect was found to be mediated through SERCA2-dependent restoration of calcium homeostasis (Ren et al., 2024). Furthermore, the Nrf2/HO-1 pathway also negatively regulates ROS-dependent NLRP3 activation through its antioxidative properties (Liu Z. et al., 2023). Consistent with this, puerarin attenuates Aβ_1–40_-induced NLRP3 activation by mitigating oxidative stress and ER stress, likely via Nrf2/HO-1 pathway activation (Wang et al., 2017b).

Degradative autophagy plays a suppressive role in restricting hyperactivation of the NLRP3 inflammasome. In the context of AMD, this pathway is frequently compromised due to the lifelong burden of phagocytosing POS and the accumulation of lipofuscin in RPE lysosomes (Kaarniranta et al., 2023; Datta et al., 2017). In IL-1α-primed ARPE-19 cells, NLRP3 inflammasome activation is induced by the proteasome inhibitor MG-132 and the autophagy inhibitor BafA, which artificially mimic the age-related impairment in intracellular clearance mechanisms. Conversely, resveratrol and omega-3 fatty acids can not only promote autophagy, but also inhibit NF-κB translocation. As a result, Resvega, their combined formulation, has been shown to alleviate NLRP3 inflammasome activation effectively, restrict caspase-1 activity, and reduce the secretion of IL-1β (Bhattarai et al., 2021).

In sum, these findings suggest that indirect modulation of upstream stress and inflammatory pathways represents an alternative strategy for restricting NLRP3 activation in AMD. However, the therapeutic effects of these interventions are often pleiotropic and cannot be solely attributed to targeted inflammasome inhibition. Because these drugs impact broad, systemic networks, their direct relevance and safety in localized retinal disease remain a translational challenge. Therefore, further studies are required to clarify the relative contribution of NLRP3 suppression to their observed protective effects and to separate targeted ocular benefits from generalized systemic anti-inflammation.

### 4.3 Modulations of extracellular vesicles

EVs are nanosized membrane-surrounded vesicles released by cells. Based on biogenesis, EVs are classified into three main subgroups: exosomes, microvesicles, and apoptotic bodies, which are derived from multivesicular endosomes, plasma membrane shedding, and membrane blebbing during apoptosis, respectively (Chen, 2024).

RPE cells enhance the release of EVs under stress, either from the apical or the basal side. These EVs carry pro-inflammatory factors, pro-angiogenic factors, and well-known drusen components such as complement proteins, Aβ, Apo E, and vitronectin, all of which may contribute to the progression of AMD (Hyttinen et al., 2025). The pathological EVs can activate the NLRP3 inflammasome. For example, high-risk RPE cells with *CFH* Y402H polymorphism were shown to release significantly more apical EVs. The uptake of these apical EVs by healthy RPEs can activate calpain that mediates the cytoskeleton disruption, which is potentially linked to pyroptotic cell death and inflammasome activation (Kurzawa-Akanbi et al., 2022; Davis et al., 2019). Another study has also demonstrated that ARPE-19 cells exposed to photo-oxidative blue-light stimulation could release exosomes, which directly upregulated the NLRP3 inflammasome (Zhang et al., 2019).

Inspired by these studies, future therapeutic strategies may target the pathological EVs. First, since the increased secretion of pathological EVs may represent a compensatory mechanism of impaired autophagy (Baixauli et al., 2014), boosting degradative autophagy by agonists or upregulating the expression of autophagy-related genes could be effective in reducing EVs release. Second, it is possible that targeting specific proteins on the surface of the pathological EVs with antibodies may facilitate their removal and disrupt their function (Nishida-Aoki et al., 2017).

While the EVs secreted by high-risk RPE cells activate inflammasomes and promote AMD progression, exosomes derived from mesenchymal stem cells (MSC-Exos) have been utilized in treating several ocular inflammatory diseases in animal models (An et al., 2025). This is ascribed to the ability of MSC-Exos to easily bypass the BRB and blood-aqueous barriers due to their lipid bilayer envelope and nanoscale size (Harrell et al., 2022). The MSC-Exos carry immunosuppressive cytokines such as TGF-β and IL-10 and miRNA such as miR-233 and miR-146a, which are all efficient in restricting NLRP3 inflammasome activation and reducing the production of IL-18 and IL-1β (Harrell et al., 2023). In a SI-triggered RPE cell degeneration model, co-culture with MSCs remarkably protected the RPE cells from inflammatory damage and cell death, which is mainly accomplished by downregulating NF-κB-mediated NLRP3 inflammasome activation and reducing ROS through maintaining mitochondrial integrity (Mao et al., 2018). Thus, it is promising that MSC-based therapies may be applied in treating AMD.

### 4.4 Translational challenges and future directions for NLRP3-targeted therapy

Despite the clinical potential of NLRP3-targeted therapies in mitigating AMD progression, several hurdles regarding their translational development must be addressed, primarily involving drug delivery strategies, tissue specificity, and long-term safety profiles.

First, as the ocular surface barriers and the BRB may decrease bioactivity of topical eye drops and systemic administration, intraocular injection is still the most commonly used modality for AMD (Urtti, 2006). Up to now, FDA-approved AMD therapeutic agents, targeting primarily VEGF and complementary pathways, are all delivered exclusively by direct intravitreal injection (Rodriguez-Cruz et al., 2025). To overcome the burden of frequent injections and enhance patient compliance, advanced delivery platforms such as sustained-release intravitreal implants, nanoparticle hydrogels, viral vectors, and exosome-based vehicles are being actively explored (Kim and Woo, 2021; Bairagi et al., 2025). These delivery systems may be particularly advantageous for NLRP3-targeted therapies.

Second, achieving high tissue and cell-type specificity is critical to avoid non-specific cytotoxicity in adjacent healthy retinal neurons. NLRP3 inflammasome activation has been identified in multiple retinal cell types, including RPE cells, microglia, infiltrating macrophages, and choroidal endothelial cells. Current cell-specific delivery strategies for AMD primarily rely on engineered viral vectors, particularly AAVs, and nanoparticle systems. By utilizing serotype engineering, cell-specific promoters, ligand-receptor recognition, and passive tissue retention, these platforms achieve precise targeting (Koo et al., 2018; Li et al., 2026; Cao et al., 2023; Himawan et al., 2019). Consequently, they maximize therapeutic delivery to key retinal populations involved in disease, including photoreceptors, RPE cells, microglia, and other disease-relevant retinal cells.

Third, beyond efficacy, evaluating the long-term safety profiles of NLRP3 inflammasome-targeted interventions remains a paramount concern for their clinical translation in AMD. Although excessive NLRP3 activation contributes to chronic retinal inflammation, physiological inflammasome signaling is also indispensable in host defense and tissue homeostasis (Honda et al., 2023). Sustained blockade may therefore increase the susceptibility to infection or impair normal immune surveillance (Vande Walle and Lamkanfi, 2024). Furthermore, impaired clearance of damaged or senescent cells might itself become a pathogenic state, as survived CECs rescued by ANXA1 can acquire a pro-angiogenic phenotype and subsequently contribute to CNV development (Zhu et al., 2022). Therefore, implementing periodic therapeutic windows, dosage titrations, or utilizing microenvironment-responsive drug delivery systems will be vital to achieve optimal therapeutic benefits while preserving baseline physiological functions and ensuring long-term ocular safety.

In summary, addressing these challenges will not only deepen our understanding of AMD pathogenesis but also accelerate the development of safe, effective, and precision-based NLRP3-targeted therapies.

## 5 Conclusions

Accumulating evidence has solidified the role of the NLRP3 inflammasome as a central hub integrating microenvironmental stress, innate immune responses, and retinal degeneration in AMD. Rather than acting as a simple, uniform inflammatory switch, NLRP3 signaling operates within a highly complex, cell-type-specific, and disease-stage-dependent network. In the early stages of AMD, sustained metabolic and oxidative stressors drive NLRP3 activation primarily in RPE cells and microglia, initiating a subclinical but self-reinforcing inflammatory cascade. This process not only triggers cell-autonomous injury through pyroptosis but also amplifies chronic neuroinflammation across the retinal microenvironment via the coordinated release of DAMPs, SASP factors, and pro-inflammatory extracellular vesicles. As the disease advances, this persistent inflammatory milieu culminates in the distinct phenotypes of late-stage AMD – either accelerating extensive geographic atrophy or promoting pathologic neovascularization.

A pivotal shift in our current understanding of NLRP3-mediated pathology is the recognition of interconnected cell death pathways. Emerging data highlight that NLRP3 activation does not occur in isolation but is deeply intertwined with apoptosis, pyroptosis, and necroptosis, converging on the multifaceted process of PANoptosis. This cross-regulation suggests that targeting a single executioner pathway may trigger compensatory cell death mechanisms, thereby limiting therapeutic efficacy. Concurrently, the discovery that exosomes and other EVs serve as critical vehicles for transporting inflammatory mediators and drusen-associated components offers a novel paradigm for intercellular communication and potential biomarkers in retinal degeneration.

Despite the therapeutic promise of NLRP3 pathway inhibition, significant translational hurdles remain before these laboratory findings can be successfully integrated into clinical practice for dry AMD patients. First, the dual nature of NLRP3 – beneficial in maintaining homeostasis versus detrimental when chronically hyperactivated – demands a delicate balance; global, indiscriminate inhibition risks compromising ocular immune surveillance. Second, given the distinct cellular contributions during different disease stages, therapeutics must achieve precise spatiotemporal delivery, targeting the right cell type (e.g., RPE versus microglia) at the optimal therapeutic window. Furthermore, resolving the discrepancies between acute rodent models of oxidative stress and the chronic, decades-long pathogenesis of human AMD is imperative for improving clinical translatability.

Looking forward, the integration of single-cell multi-omics, high-resolution spatial transcriptomics, and patient-derived iPSC-RPE models will be essential to map the precise spatiotemporal landscape of NLRP3 activation. Future therapeutic strategies should pivot toward combination therapies that simultaneously inhibit the NLRP3 inflammasome and its interconnected PANoptotic pathways, or leverage engineered EVs for targeted retinal delivery. Overcoming these challenges will pave the way for personalized, mechanism-driven interventions that can successfully halt or reverse the progression of this devastating blinding disease.

## Author contributions

MZ: Writing – original draft. WY: Writing – review & editing.
